# Kystes de l'ovaire: score échographique de malignité

**DOI:** 10.11604/pamj.2014.18.215.4037

**Published:** 2014-07-15

**Authors:** Kaouther Dimassi, Hajeur Bettaieb, Mohammed Derbel, Amel Triki, Mohammed Faouzi Gara

**Affiliations:** 1 Service de Gynécologie-Obstétrique, Hôpital Mongi Slim La Marsa, Tunisie

**Keywords:** Kyste de l'ovaire, échographie, tumeur maligne, score, Ovarian cyst, echography, malignant tumor, score

## Abstract

Les kystes de l'ovaire constituent l'un des motifs les plus fréquents de consultation en gynécologie. L’étape diagnostique repose principalement sur l’échographie. Nous proposons dans ce travail un score échographique reproductible prédictif de malignité. Nous évaluons la fiabilité de ce score après confrontations des données échographiques et histologiques. Il s'agit d'une étude rétrospective réalisée sur une période de 3 ans. Nous avons élaboré un score basé sur les signes échographiques décrits dans la littérature comme prédictifs de malignité et avons classé les examens échographiques préopératoires selon leurs scores respectifs. Les données échographiques étaient comparées aux résultats histologiques et un seuil prédictif de malignité a été déterminé pour le score adopté. 150 patientes ont été colligées. Les deux signes échographiques les plus prédictifs de malignité étaient: les végétations endo-kystiques, avec une Valeur Prédictive Positive (VPP) à 86,67% et une Valeur Prédictive Négative (VPN) à 100%, et le caractère vascularisé au Doppler couleur avec une VPP à 72,52% et une VPN à 100%. Le seuil retenu pour le score proposé était de 6 avec une spécificité de 100%, une sensibilité de 100%, une VPP de 100% et une VPN de 100%. L’échographie joue un rôle décisif dans la conduite à tenir devant une masse ovarienne. Seul un faisceau d'arguments permet d’évoquer la malignité lors de l'examen échographique. L'utilisation de scores basés sur des signes simples, reproductibles augmente la valeur diagnostique de l’échographie en matière de malignité.

## Introduction

Les kystes de l'ovaire constituent l'un des motifs les plus fréquents de consultation en gynécologie. Environ 5% des femmes développent un kyste de l'ovaire au cours de leur vie, ils peuvent survenir à tout âge et sont bénins dans 95% des cas [1]. L’étape diagnostique repose principalement sur l’échographie qui permet une orientation étiologique et pronostique; elle est très fiable pour différentier le caractère fonctionnel de l'organique [1]. Cependant, le diagnostic de malignité et de bénignité est moins formel. Dans ce sens, et afin de mieux orienter la prise en charge thérapeutique; différents scores échographiques ont été proposés. Nous proposons dans ce travail un score échographique prédictif de malignité. Nous évaluons la fiabilité de ce score après confrontations des données échographiques au diagnostic anatomopathologique.

## Méthodes

Il s'agit d'une étude rétrospective réalisée au service de gynécologie obstétrique de l'hôpital Mongi Slim la Marsa, Tunisie, sur une période de 3 ans allant du 1er Janvier 2010 au 31 décembre 2012. Nous avons inclus les patientes opérées pour un kyste de l'ovaire dans un contexte d'urgence ou de chirurgie à froid et qui avaient bénéficié en préopératoire d'un examen échographique détaillé et documenté par une iconographie. Nous avons exclu de cette étude, les patientes non opérées dans notre unité. Nous avons établi une fiche pour chaque patiente afin de réunir les caractéristiques épidémiologiques, les résultats biologiques ainsi que le résultat de l'analyse histologique du kyste. Nous avons élaboré un score noté sur 8 basé sur les signes échographiques décrits dans la littérature comme étant évocateurs de malignité [2] (Tableau 1). Ainsi, les signes échographiques utilisés pour calculer le score étaient: La taille supérieure à 7cm, la présence de végétations endokystiques, la multilocularité ou la présence de cloisons épaisses, le caractère richement vascularisé au Doppler couleur, la présence d'un épanchement intrapéritonéal, et le caractère bilatéral de l'atteinte. Les comptes rendus échographiques ainsi que les clichés iconographiques ont été repris et les différents signes échographiques suscités recherchés. On avait accordé un point chaque fois qu'un signe du score était objectivé. Un score était ainsi calculé pour chaque examen échographique sur un total de 8 (Tableau 1). Par la suite, le résultat de l'examen anatomopathologique du kyste était systématiquement comparé aux scores échographiques. Toutes les données étaient recueillies sur Epi info 6, et l'analyse statistique était réalisée par le logiciel SPSS 15.0. L’étude de la corrélation de chaque signe échographique au diagnostic histologique de malignité a été effectuée par le test de chi-deux de Pearson. Le seuil de signification a été fixé à 0,05. Nous avons utilisé la courbe *Receiver Operating Characteristic Curve* (ROC) pour déterminer la valeur seuil du score échographique à partir de laquelle le diagnostic de malignité peut être fortement suspecté.

**Tableau 1:** Score échographique de malignité utilisé dans l’étude

	Présent	Absent
Taille supérieure à 7cm	1point	0 point
Paroi supérieure à 4mm	1point	0 point
Composante hétérogène (solido-kystique)	1point	0 point
Présence d'au moins une végétation endokystique	1point	0 point
Multilocularité ou cloisons épaisses	1point	0 point
Tumeur vascularisée au Doppler couleur. (paroi, cloison ou végétation)	1point	0 point
Présence d'un épanchement intra péritonéal	1point	0 point
Atteinte bilatérale	1point	0 point
TOTAL	08 points	0 points

## Résultats

Nous avons colligé 150 patientes opérées pour un kyste de l'ovaire durant la période d’étude. L’âge moyen des patientes était de 36,6 ans avec des extrêmes allant de 15 à 58 ans. Les caractéristiques épidémiologiques de nos patientes sont résumées dans leTableau 2. Les motifs de consultations étaient essentiellement des douleurs pelviennes (68% des cas). Les kystes étaient de découverte fortuite dans 13,3% des cas. Dans 10% des cas; le diagnostic était posé au cours d'un bilan d'infertilité.

**Tableau 2:** Caractéristiques épidémiologiques des patientes de l’étude

	Nombre	Pourcentage
Antécédents familiaux: tumeur de l'ovaire ou du sein	17	11,3
Antécédents personnels de kyste de l'ovaire	28	18,6
Ménopause	19	12,6
Antécédents d'infertilité	15	10
Autres antécédents chirurgicaux	37	24,6
Grossesse en cours	9	6

**Figure 1:**
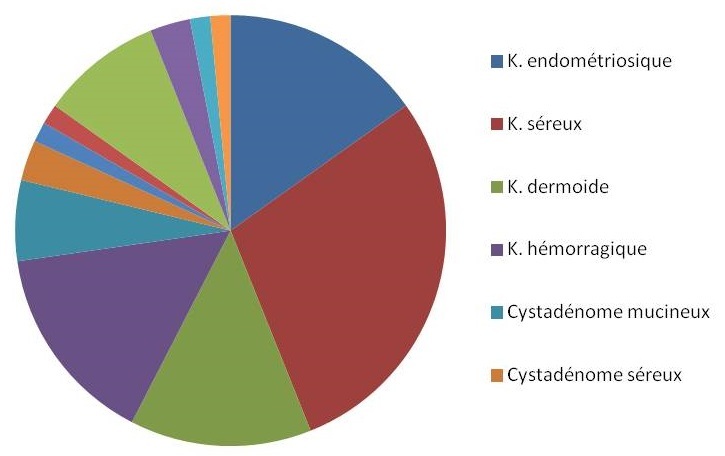
Différents types histologiques des tumeurs organiques de l'ovaire

L'examen anatomopathologique avait mis en évidence une pathologie maligne dans 9 cas (6% des cas). L'atteinte histologique la plus fréquente était le carcinome papillaire séreux: 6/9 cas. Ailleurs, l'examen anatomopathologique avait conclu à un carcinome mucineux dans 2 cas, et à un tératome malin dans un cas. Deux cas de tumeurs à la limite de la malignité (Borderline) ont été notés (Figure 1).

**Figure 2:**
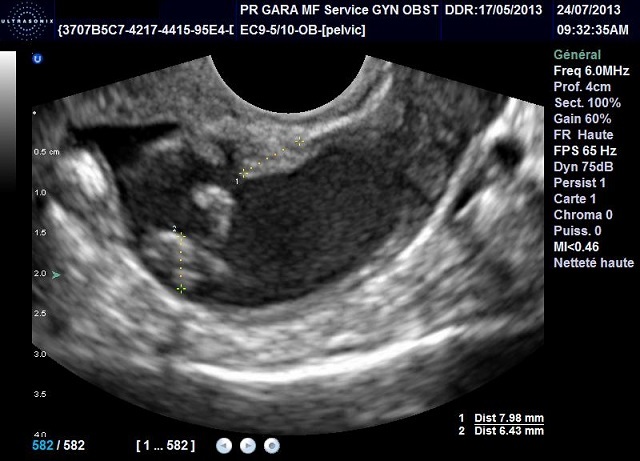
Aspect échographique de végétations endokystiques

**Figure 3:**
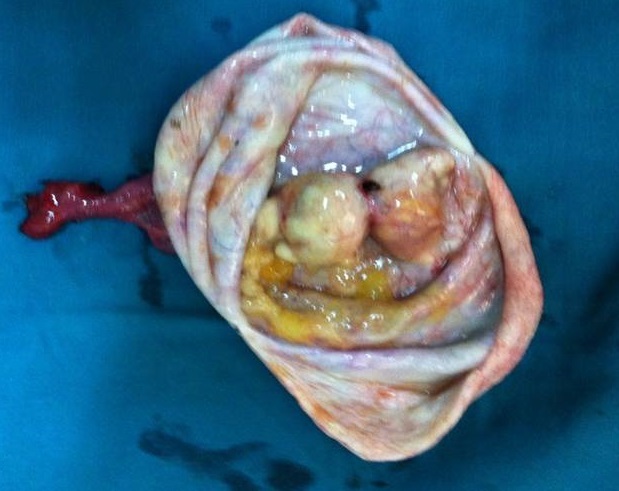
Aspect macroscopique de végétations endokystiques

Dans un premier temps, nous avons étudié chaque signe échographique utilisé pour le calcul du score et avons évalué sa corrélation avec le diagnostic histologique de malignité (Tableau 3). Ainsi, une relation significative (seuil < 0.05) était objectivée pour les signes échographiques suivants: végétations endokystiques, vascularisation au Doppler couleur, multilocularité, épanchement péritonéal, composante hétérogène, paroi épaisse et le caractère bilatéral. De plus, les deux signes échographiques les plus corrélés au diagnostic de malignité dans notre étude étaient: les végétations endo-kystiques (Figure 2, Figure 3), avec une Valeur Prédictive Positive à 86,67% et une Valeur Prédictive Négative à 100%, et le caractère vascularisé au Doppler couleur avec une VPP à 72,52% et une VPN à 100%.

**Tableau 3:** Corrélations des différents signes échographiques avec le diagnostic histologique de malignité

	VPP	VPN	Sensibilité	Spécificité	p
Taille supérieure à 7cm	21%	96%	55%	58%	0,48
Epaisseur paroi supérieure à 4mm	20%	99%	100%	58%	0,001
Composante hétérogène solido-kystique	21,5%	96%	88%	71%	0,001
Végétation endokystique	86,6%	100%	100%	92%	<0,0001
Multilocularité	34%	100%	100%	74%	<0,0001
Vascularisation au Doppler couleur	72,52%	100%	100%	96%	<0,0001
Epanchement péritonéal	61,2%	100%	100%	92%	<0,0001
Atteinte bilatérale	67%	97,5%	33%	97%	0,005

VPN: Valeur prédictive négative. VPP: Valeur prédictive positive

**Figure 4:**
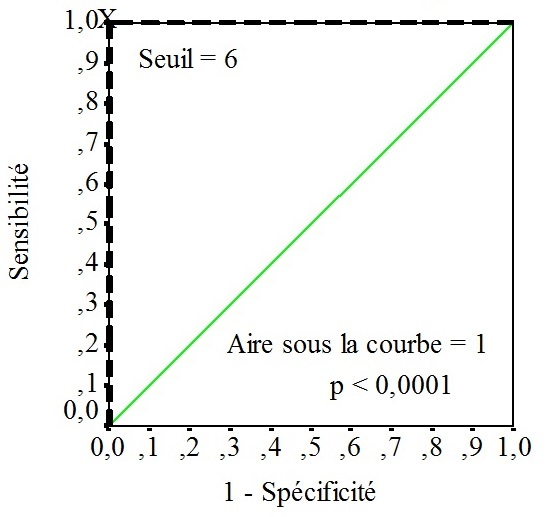
Seuil prédictif de malignité selon la courbe ROC

Dans un deuxième temps, nous avons utilisé la courbe ROC pour déterminer la valeur seuil du score à partir de laquelle le diagnostic de malignité peut être fortement suspecté (Figure 4). Le seuil retenu était de 6/8. Ce seuil avait une spécificité de 100%, une sensibilité de 100%, une VPP de 100% et une VPN de 100%.

**Figure 5:**
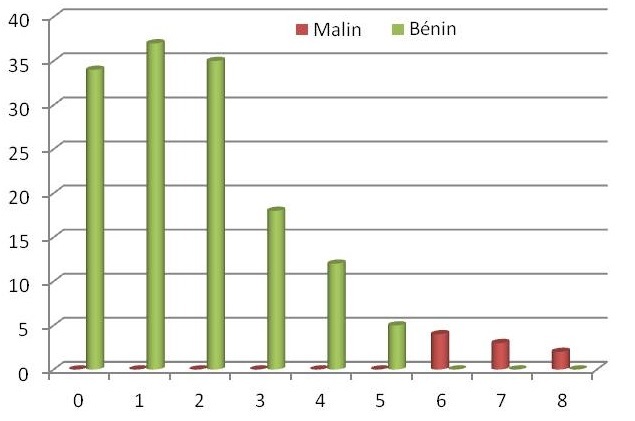
Répartition des tumeurs ovariennes selon le type histologique (bénin/malin) et le score échographique

Enfin, la Figure 5 représente la répartition des différentes tumeurs selon le score échographique (de 00 à 08) et le type histologique: bénin/malin.

## Discussion

La découverte fortuite d'une masse annexielle est une éventualité de plus en plus fréquente en raison du développement des techniques d'imagerie et en particulier de l’échographie. La prise en charge de ces masses annexielles va de la kystectomie simple, voire même de l'abstention, à la laparotomie médiane avec hystérectomie totale non conservatrice, omentectomie, curage iliaque et lomboaortique en cas de cancer. L'attitude devant une masse annexielle asymptomatique va dépendre de la clinique, du statut hormonal en regard de la ménopause et du désir de grossesse, de l'histoire carcinologique familiale et personnelle, des marqueurs tumoraux biologiques, et en particulier de l'imagerie. L’échographie, examen d'imagerie clé pour l'ovaire, apparaît simple de réalisation et d'interprétation, mais nécessite un apprentissage rigoureux et l'acquisition d'une solide expérience. Lors de la découverte d'une masse pelvienne, l’échographie doit faire le diagnostic positif (tumeur ovarienne), en éliminant les diagnostics différentiels (tumeurs pelviennes non ovariennes), puis le diagnostic d'organicité en éliminant les kystes fonctionnels, ensuite le diagnostic du type de tumeur et enfin le diagnostic de malignité en recherchant tout signe suspect. Il est capital de pouvoir renseigner le chirurgien pour prendre les décisions suivantes: (1) Faut-il opérer? Dans quels délais? (2) Par quelle voie (cœlioscopie ou laparotomie)? Dans quel hôpital? (3) Faut-il réaliser une annexectomie ou une kystectomie?

Il est possible d'utiliser l'analyse morphologique d'une image échographique pour distinguer certains types de tumeurs: c'est la *pattern recognition* [2]. En effet, un échographiste averti cherche les mêmes informations que le chirurgien ou le pathologiste lorsqu'il sélectionne la tumeur pour en observer l'intérieur. De nombreuses masses pelviennes ont une apparence macroscopique typique et un diagnostic fiable peut être évoqué sur la base de cette apparence et donc sur la base de données échographiques [2].

Dans notre étude, nous avons utilisé cette analyse morphologique des images et avons axé notre approche sur la recherche de signes échographiques suspects de malignité. Ainsi, les deux signes échographiques les plus corrélés à la malignité étaient les végétations endo-kystiques et l'aspect vascularisé au Doppler couleur.

Sur le plan histologique, les végétations résultent d'excroissances constituées d'un stroma de densité variable, recouvert d'un épithélium uni stratifié (Figure 3). Dans la littérature, le risque de malignité associé à cette particularité varie de 40,5 à 83,7% [3]. Il est d'autant plus grand que le nombre de végétations est plus grand [3]. Dans notre série les images kystiques avec au moins une végétation endokystique avaient une VPP de malignité de 86, 6% et une VPN de 100%. Nous n'avons pas précisé le nombre de végétations visualisées afin de faciliter l'examen échographique. En effet, ces végétations ne sont pas toujours facilement identifiées à l’échographie. Il ressort de l'ensemble des études que l’échographie vaginale a une sensibilité dans la détection des végétations variant de 69 à 98% et une spécificité de 52 à 91,2%, une valeur prédictive positive de 37 à 60% et une valeur prédictive négative de 92,6 à 100%.

L'analyse Doppler d'une masse ovarienne est semi-quantitative. Elle permet une appréciation subjective de la localisation de la vascularisation et de son intensité à l'aide du Doppler couleur ou énergie (Power Doppler). Un flux doit être recherché au niveau de la paroi de la tumeur, de ses éventuelles cloisons ou zones solides. Il est établi que la vascularisation des tumeurs bénignes est plutôt périphérique et que celle des cancers est centrale [2]. Cependant, La vascularisation des cloisons ou septas n'est pas spécifique de malignité. Elle peut être retrouvée dans certaines tumeurs bénignes à septa comme les tumeurs mucineuses et dans les abcès tubo-ovariens [4]. Dans notre étude, la VPP de ce signe était de 72,52%.

D'autres signes échographiques ont été décrits avec une moins bonne corrélation avec le diagnostic de malignité [2]. En exemple, la multilocularité, qui caractérise la division d'un kyste en compartiments par des cloisons, est liée à une atteinte maligne dans 18 à 37% des cas [5]. Dans notre étude, ce signe était significativement corrélé au diagnostic de malignité avec une sensibilité de 100% et une spécificité de 74%. De même, la présence d'une paroi épaisse (supérieure à 4mm) [3] ne signe pas toujours la malignité, puisque ce signe est aussi retrouvé en cas de tératome ou d'endométriome. Dans notre série, ce signe échographique avait une faible spécificité de 58%. Enfin, en dépits du fait que le risque de cancer augmente avec la taille de la masse ovarienne [5], ce signe reste peut fiable pour faire évoquer la malignité avec un risque de cancer allant de 7 à 35,6% pour une taille comprise entre 5 et 10 cm et de 12,5 à 71,8% pour un kyste mesurant plus de 10 cm [5]. Dans notre série, une taille supérieure à 7cm n’était pas significativement corrélé au diagnostic de malignité (p = 0.48). Alcazar et al. [6] ont utilisé une régression logistique dans une série de 665 tumeurs pour distinguer les facteurs prédictifs de malignité. L'analyse uni-variée a montré que tous les critères précédemment cités étaient statistiquement significatifs. Mais c'est une analyse multivariée qui a permis de faire ressortir les critères qui étaient indépendants: présence de végétation et composante solide. Par exemple, le paramètre « cloison épaisse » de l’étude de Sassone [7] et le paramètre « volume » proposé par De Priest [8] ne sont pas vraiment des facteurs prédictifs indépendants. De même dans notre étude, considérés un à un ces deux paramètres sont peu prédictifs de malignité.

Actuellement, seul un faisceau d'arguments permet d’évoquer la malignité. L'analyse uni-variée de la plus large étude sur le sujet montre que les paramètres échographiques choisis permettent tous d'aider à différencier le bénin du malin, mais qu'aucun ne peut le faire seul. Les paramètres échographiques en faveur de la malignité classiquement admis sont: [9] Le volume de la tumeur; La bilatéralité de l'atteinte; Une paroi d’épaisseur supérieure ou égale à 3mm; La présence de végétations endokystiques; Une cloison intra-kystique égale ou supérieure à 3mm ou une aire solide; La présence d’épanchement intra péritonéal.

Le risque de cancer augmente d'autant plus lorsque ces signes sont associés [2]. Ainsi, a été introduite l'idée d'utiliser des scores morphologiques pour relever correctement le challenge du diagnostic préopératoire des tumeurs ovariennes [4, 9]. Dans ce sens, un kyste uniloculaire à paroi fine et lisse a un score bas, alors qu'un kyste vascularisé, multiloculaire à paroi épaisse et à contenu hétérogène a un score élevé. Dans cette étude, nous présentons un score échographique pur, utilisant des signes morphologiques simples et reproductible. L'idée étant d'amener les opérateurs à réaliser une étude complète du kyste en recherchant systématiquement tous les signes évocateurs de malignité. Nos résultats, retiennent le seuil de 06 sur 08 pour évoquer fortement la malignité avec une sensibilité de 100%, une spécificité de 100%, une VPN de 100% et une VPP de 100%. Les scores échographiques utilisés sont nombreux dans la littérature et obtiennent généralement de bons résultats [6–8]. Ainsi, une masse annexielle avec un score échographique supérieur ou égal à 9 est considérée maligne selon le score de Sassone [7]. Timmerman et al [10], utilisent aussi un score échographique pur basé sur des signes échographiques simples: tumeur solide irrégulière; présence d'un épanchement péritonéal; présence d'au moins de quatre végétations; tumeur irrégulière multi cloisonnée solide dont un diamètre est supérieur à 10 cm et un score colorimétrique fort. Nous rappelons que dans le score utilisé dans notre étude, le nombre de végétations n'est pas précisé. En effet, nous avons constaté que la présence d'au moins une végétation avait une bonne corrélation au diagnostic de malignité (VPP = 86.6%, VPN = 100%). De plus, l’échographie endovaginale ayant une sensibilité et une spécificité variables et opérateur-dépendantes en matière de végétation, nous avons opté vers l’élaboration d'un score reproductible et accessible aux échographistes de niveau d'expérience variable. Enfin, une étude prospective s'avère nécessaire avec l’élaboration préalable des différents critères du score. Dans la littérature, d'autres paramètres échographiques ont été intégrés dans les calculs de score de malignité. En exemple, certaines équipes utilisent le Doppler pulsé pour mesurer des critères hémodynamiques [8]. Les publications initiales sur l'utilisation du Doppler couleur et pulsé dans l'amélioration du diagnostic de malignité étaient encourageantes, mais les études suivantes ont mis en doute ces si bons résultats. La mesure de l'index de résistance (IR) semble plus discriminante que l'index de pulsatilité. Un IR bas (inférieur à 0.4, à 0.45 ou à 0.53 selon les auteurs [2] est en faveur de la malignité, mais non pathognomonique. Ces mesures hémodynamiques, en dépits de leurs limites, restent intéressantes pour la constitution d'un faisceau d'arguments dans la discrimination malin/bénin. Leur réalisation nécessite une courbe d'apprentissage et il serait intéressant de les intégrer dans le calcul d'un score échographique en prospectif.

Il est clair que l'utilisation des scores a pour objectif de faire évoquer la possibilité d'un cancer en préopératoire, mais aussi de l’éliminer notamment en cas d'image simple. Les images simples sont peu volumineuses (moins de 5 cm selon la plupart des auteurs), uniloculaires, anéchogènes pures (liquidiennes), sans végétation, ni zone solide, à cloison fine inférieure à 3mm ou sans cloison. Elles sont quasiment toutes bénignes, le risque de malignité est de l'ordre de 3’ dans la littérature [2]. Dans notre étude aucune image simple avec un score bas ne s’était avérée être maligne (Figure 5).

Enfin, les scores utilisés dans la littérature ne sont pas toujours échographiques stricts, et un bon nombre tient compte des caractéristiques épidémiologiques ou biologiques de la patiente [9]. En exemple, le « Risk Malignancy index », décrit par l’équipe de Jacobs en 1990 [11], fait encore l'objet de nombreuses séries [2]. Ce score associe des critères échographiques, le dosage du Ca125, et le statut ménopausique. Le score utilisé dans notre étude était strictement échographique. Il est évident que l'on ne peut pas se contenter d'additionner toutes les caractéristiques morphologiques pour décider d'une intervention, une telle addition pouvant conduire à des résultats faussement positifs [3]. Mais le but de l’étude était d’étudier la fiabilité de l’échographie seule en tant qu'outil d'imagerie, indépendamment du tableau clinique.

## Conclusion

L’échographie joue un rôle décisif dans la conduite à tenir devant une masse ovarienne. En effet, elle doit faire le diagnostic positif, puis le diagnostic d'organicité, ensuite le diagnostic du type de tumeur et enfin le diagnostic de malignité en recherchant tout signe suspect. Actuellement, seul un faisceau d'arguments permet d’évoquer la malignité lors de l'examen échographique. L'utilisation de scores basés sur des signes simples, reproductibles et utilisables pour tous comme le score proposé dans cette étude est fiable et augmente la valeur diagnostique de l’échographie en matière de malignité.
